# Fully Bayesian Prediction Algorithms for Mobile Robotic Sensors under Uncertain Localization Using Gaussian Markov Random Fields

**DOI:** 10.3390/s18092866

**Published:** 2018-08-30

**Authors:** Mahdi Jadaliha, Jinho Jeong, Yunfei Xu, Jongeun Choi, Junghoon Kim

**Affiliations:** 1 Monsanto, St. Louis, MO 63146, USA; jadaliha@gmail.com; 2 School of Mechanical Engineering, Yonsei University, Seoul 03722, Korea; jinhojeong@yonsei.ac.kr; 3 Denso International America, Inc., San Jose, CA 95110, USA; feiyun.xu@gmail.com; 4 Department of Civil and Environmental Engineering, Yonsei University, Seoul 03722, Korea; junghoon@yonsei.ac.kr

**Keywords:** Gaussian markov random field, fully Bayesian, mobile sensor network, localization uncertainty

## Abstract

In this paper, we present algorithms for predicting a spatio-temporal random field measured by mobile robotic sensors under uncertainties in localization and measurements. The spatio-temporal field of interest is modeled by a sum of a time-varying mean function and a Gaussian Markov random field (GMRF) with unknown hyperparameters. We first derive the exact Bayesian solution to the problem of computing the predictive inference of the random field, taking into account observations, uncertain hyperparameters, measurement noise, and uncertain localization in a fully Bayesian point of view. We show that the exact solution for uncertain localization is not scalable as the number of observations increases. To cope with this exponentially increasing complexity and to be usable for mobile sensor networks with limited resources, we propose a scalable approximation with a controllable trade-off between approximation error and complexity to the exact solution. The effectiveness of the proposed algorithms is demonstrated by simulation and experimental results.

## 1. Introduction

In recent years, there has been an increasing exploitation of mobile robotic sensors in environmental monitoring [1,2]. Gaussian processes defined by mean and covariance functions over a continuum space have been frequently used for mobile sensor networks to statistically model physical phenomena such as harmful algal blooms, pH, and temperature, e.g., [3,4,5]. The significant computational complexity in Gaussian process regression due to the growing number of observations has been tackled in different ways. Xu et al. [4] analyzed the conditions under which near-optimal prediction can be achieved, using truncated observations when the covariance function is known a priori. Xu and Choi [6] developed a new efficient and scalable inference algorithm for a class of static Gaussian processes that builds on a Gaussian Markov random field (GMRF) is developed for known hyperparameters. In terms of the computational cost reduction, Ref. [7,8] showed that Gaussian processes can be formulated as infinite-dimensional Kalman filtering and such approach can scale down computational complexity. Ref. [9] combined Gaussian process and Kalman filter for efficient computation. On the other hand, unknown hyperparameters in the covariance function can be estimated by a maximum likelihood (ML) estimator or a maximum a posteriori (MAP) estimator and then can be used for the prediction [10]. However, the point estimate itself needs to be identified using a certain amount of measurements and it does not fully incorporate the uncertainty in the estimated hyperparameters into the prediction in a Bayesian perspective.

The advantage of a fully Bayesian approach is the capability of incorporating various uncertainties in the model parameters and measurement processes in the prediction [2]. However, the solution often requires an approximation technique such as Markov Chain Monte Carlo (MCMC), Laplace approximation, or variational Bayes methods, which still requires a high level of computational complexity [2]. Xu et al. [11] designed a sequential Bayesian prediction algorithm and its distributed version for Gaussian processes to deal with uncertain bandwidths. Xu et al. [12] presented sequential fully Bayesian prediction algorithms for a static GMRF with unknown hyperparameters.

Inexpensive wireless/mobile sensor networks [13] are widespread at the cost of precision in localization. Due to their growing usage, there are many practical opportunities where continuously sampled measurements need to be fused for sensors with localization uncertainty [14]. Secure indoor localization has been proposed based on extracting trusted fingerprint [15]. Theoretically-correct yet efficient (or scalable) inference algorithms need to be developed to meet such demands.

Related works involving uncertain localization in our context are as follows. Gaussian process regression has been used in building maps and localization in many practical applications. Brooks et al. [16] utilized Gaussian process regression to model geo-referenced sensor measurements (obtained from a camera) in a supervised learning manner. Kemppainen et al. [17] used Gaussian process regression to implement simultaneous localization and mapping (SLAM) using a magnetic field. O’Callaghan et al. [18] investigated the problem of using laser range-finder data to probabilistically classify a robot’s environment. McHutchon and Rasmussen [19] presented a Gaussian process for training on input points corrupted by independent and identically distributed (i.i.d.) Gaussian noise. Do et al. [20] conducted visual feature selection via Gaussian process regression for position estimation using an omnidirectional camera. The works in [19,20] assume that all hyperparameters are trained offline a priori. Jadaliha et al. [21] and Choi et al. [22] formulated and solved the problem of Gaussian process regression with uncertain localization and known hyperparameters for both centralized and distributed fashions, respectively. A key limitation of such approaches with fixed hyperparameters arises from the fact that, after the initial training phase, learning is discontinued. If the environment changes, it is desirable that the localization algorithm adapts to the changes on the fly. A fully Bayesian approach that treats hyperparameters as random variables can address this issue with increased computational complexity.

The novelty of our work in contrast to ones in [11,12] is to fully consider the uncertainty on the sampling positions along with other uncertainties such as hyperparameters, observation noise, etc., in a *fully Bayesian* manner. The fully Bayesian field SLAM presented in [23] is quite similar to our current work in this paper. However, it is limited to a static random field while ours can deal with the time-varying random field. To the best of our knowledge, fully Bayesian prediction algorithms for spatio-temporal random fields that can take into account uncertain localization are scant to date. Hence, this paper aims to develop such inference algorithms for robotic sensor networks in practical situations by building on spatio-temporal models developed by Lynch et al. [1] and Xu et al. [2]. With continuous improvement in computation power in embedded systems, it is very important to prepare theoretically-correct, and flexible fully Bayesian approach to cope with such practical problems.

The contributions of the paper are as follows. Firstly, we model a physical spatio-temporal random field as a GMRF with uncertain hyperparameters and formulate the prediction problems with and without localization uncertainty. Next, we derive the exact Bayesian solution to the problem of computing the predictive inference of the random field, taking into account uncertain hyperparameters, measurement noise, and uncertain localization in a fully Bayesian point of view. We show that the exact solution for uncertain localization is not scalable as the number of observations increases. To cope with this increasing complexity, we propose a scalable approximation with a controllable trade-off between approximation error and complexity to the exact solution. The effectiveness of the proposed algorithms is demonstrated by experimental results in both static and dynamical environments.

The paper is organized as follows. In Section 2, we explain how a GMRF can be viewed as a sparse and discretized version of a Gaussian process. In Section 3 and Section 4, we introduce a spatio-temporal field model based on a GMRF and the mobile sensor network. In Section 5.1, we present a fully Bayesian inference approach to estimate the spatio-temporal field. The Bayesian prediction algorithm is extended for uncertain sampling positions in Section 5.2. Finally, we evaluate our approach on a real experimental setup in Section 6.

Standard notation is used. Let R and $Z_{>0}$ denote, respectively, the sets of real and positive integer numbers. The operator of expectation is denoted by E. A random vector *x*, which has a multivariate normal distribution of mean vector μ and covariance matrix Σ, is denoted by x∼N(μ,Σ). For given G={c,d} and H={1,2}, the multiplication between two sets is defined as H×G={(1,c),(1,d),(2,c),(2,d)}. Other notation will be explained in due course.

## 2. From Gaussian Processes to Gaussian Markov Random Fields

There are efforts to fit a computationally efficient GMRF on a discrete lattice to a Gaussian random field on a continuum space [12,24,25]. It has been demonstrated that GMRFs with small neighborhoods can approximate Gaussian fields surprisingly well [24]. This approximated GMRF and its regression are efficient and very attractive [26] as compared to the standard Gaussian process and its regression. Fast kriging of large data sets by using a GMRF as an approximation of a Gaussian random field has been proposed in [25].

We now briefly review a GMRF as a discretized Gaussian process on a lattice. Consider a zero-mean Gaussian process: $z\left(q\right)∼\mathrm{GP}(0,\Sigma \left(q,q^{\prime }\right)),$ where Σ(·,·) is the covariance function defined in a continuum space $S_{c}$. We discretize the compact domain $S_{c}:=\left[0\;x_{max}\right]\times \left[0\;y_{max}\right]$ into *n* spatial sites $S:=\left\{s^{\left[1\right]},\cdots ,s^{\left[n\right]}\right\}\subset R^{2}$, where $n=hx_{max}\times hy_{max}$. *h* will be chosen such that $n\in Z_{>0}$. Note that n→∞ as h→∞. The collection of realized values of the random field in S is denoted by $z:={\left(z^{\left[1\right]},\cdots ,z^{\left[n\right]}\right)}^{T}\in R^{n}$, where $z^{\left[i\right]}:=z\left(s^{\left[i\right]}\right)$.

The prior distribution of *z* is given by $z∼N(0,\Sigma_{0})$, and so we have

(1) $$\pi \left(z\right)\propto \exp\left(-\frac{1}{2}z^{T}\Sigma_{0}^{-1}z\right),$$

where $\Sigma_{0}\in R^{n\times n}$ is the covariance matrix. The i,j-th element of $\Sigma_{0}$ is defined as $\Sigma_{0}^{\left[ij\right]}=\mathrm{Cov}\left(z^{\left[i\right]},z^{\left[j\right]}\right)=\Sigma \left(z^{\left[i\right]},z^{\left[j\right]}\right)$. The prior distribution of *z* can be written by a precision matrix $Q_{0}=\Sigma_{0}^{-1}$, i.e., $z∼N(0,Q_{0}^{-1})$. This can be viewed as a discretized version of the Gaussian process (or a GMRF) with a precision matrix $Q_{0}$ on S. Note that $Q_{0}$ of this GMRF is not sparse. However, a sparse version of $Q_{0}$, i.e., ${\hat{Q}}_{0}$ with a local neighborhood that can represent the original Gaussian process can be found, for example, making ${\hat{Q}}_{0}$ close to $Q_{0}$ in some norm [24,25]. This approximate GMRF will be computationally efficient due to the sparsity of ${\hat{Q}}_{0}$. For our main problems, we will use a GMRF with a sparse precision matrix that represents a Gaussian process precisely.

We assume that we take *N* noisy measurements $y={\left(y^{\left[1\right]},\cdots ,y^{\left[N\right]}\right)}^{T}\in R^{N}$ from corresponding sampling locations $q_{c}={\left(q_{c}^{[1]T},\cdots ,q_{c}^{[N]T}\right)}^{T}\in S_{c}^{N}$. The measurement model is given by

(2) $$y^{\left[i\right]}:=y\left(q_{c}^{\left[i\right]}\right)=z\left(q_{c}^{\left[i\right]}\right)+\epsilon^{\left[i\right]},\forall i=1,\cdots ,N,$$

where $\epsilon^{\left[i\right]}\overset{i.i.d.}{∼}N\left(0,\sigma_{\epsilon }^{2}\right)$ is the measurement noise and is assumed to be independent and identically distributed (i.i.d.).

Using Gaussian process regression, the posterior distribution for $z\in R^{n}$ is given by

(3) $$z|q_{c},y∼N\left(\mu ,\Sigma \right).$$

The predictive mean $\mu \in R^{n}$ and covariance matrix $\Sigma \in R^{n\times n}$ can by obtained by $\mu =K^{T}C^{-1}y,\;\Sigma =\Sigma_{0}-K^{T}C^{-1}K,$ where the covariance matrices are defined as $K:=\mathrm{Cov}\left(y,z\right)\in R^{N\times n}$, $C:=\mathrm{Cov}\left(y,y\right)\in R^{N\times N}$, and $\Sigma_{0}:=\mathrm{Cov}\left(z,z\right)\in R^{n\times n}$.

The pose of a robot can be estimated by fusing different sensory information producing its estimate and estimation error statistics [27,28]. Throughout the paper, we assume that the positions of mobile robotic sensors and their uncertainties are estimated by a standard technique. Having had the aforementioned assumption, from a localization algorithm, the prior distribution for sampling location $q_{c}^{\left[i\right]}$ is given as $\pi (q_{c}^{\left[i\right]}|{\tilde{q}}_{c}^{\left[i\right]})$, possibly with a compact support in $S_{c}$. Then, the predictive distribution of *z* given the measured locations ${\tilde{q}}_{c}={\left({\tilde{q}}_{c}^{[1]T},\cdots ,{\tilde{q}}_{c}^{[N]T}\right)}^{T}$ is thus given by

(4) $$\begin{array}{cc} \pi (z|{\tilde{q}}_{c},y) & =\int_{q\in S_{c}}\pi \left(z|q,y\right)\pi \left(q|{\tilde{q}}_{c},y\right)dq, \end{array}$$

where π(z|q,y) can be obtained by Equation (3). However, the predictive distribution in Equation (4) does not have a closed-form solution and needs to be computed either by MCMC methods or approximation techniques [29].

Now we consider a discretized version of the Gaussian process, i.e., (GMRF) with a precision matrix $Q_{0}$ on S. Since the sampling points of Gaussian process regression are not necessarily on a finite compact domain S, we use the nearest grid point of a given sampling point $q_{c}$ in $S_{c}$ $q^{\left[i\right]}=\arg\min_{q\in S}∥q_{c}^{\left[i\right]}-q∥.$ The sampling positions for the GMRF are then exactly on the lattice, i.e., $q^{\left[i\right]}\in S$. The posterior distribution of $z\in R^{n}$ on S given by measurements in $y\in R^{N}$ and sampling positions in $q={\left(q^{[1]T},\cdots ,q^{[N]T}\right)}^{T}\in S^{N}$ is then obtained by

(5) $$z|q,y∼N(Q^{-1}b,Q^{-1}),$$

where $Q=Q_{0}+HP^{-1}H^{T},$ $b=HP^{-1}y,$ with $P=\sigma_{\epsilon }^{2}I\in R^{N\times N}$ and $H\in R^{n\times N}$ defined as

(6) $$\begin{array}{c} H^{\left[ij\right]}=\left\{\begin{array}{cc} 1, & \mathrm{if}\;s^{\left[i\right]}=q^{\left[j\right]},\\ 0, & \mathrm{otherwise}. \end{array}\right. \end{array}$$

The proof of this posterior distribution of *z* in Equation (5) is very similar to that of Proposition 6.1 in Chapter 6 of [2], which was derived by using the Woodbury matrix identity.

We consider again localization uncertainty for this GMRF. Let the measured noisy location ${\tilde{q}}^{\left[i\right]}$ be the nearest grid point of the measured noisy sampling point ${\tilde{q}}_{c}^{\left[i\right]}$ of the Gaussian process. Now we obtain a set of discretized probabilities in S induced by the continuous prior distribution defined in $S_{c}$. The discrete prior distribution for the sampling location $q^{\left[i\right]}$ is given by

(7) $$\pi \left(q^{\left[i\right]}=s^{\left[j\right]}|{\tilde{q}}^{\left[i\right]}\right)=\int_{s\subset V_{j}}\pi \left(s|{\tilde{q}}^{\left[i\right]}\right)ds,$$

where $\pi (s|{\tilde{q}}^{\left[i\right]})$ is the continuous prior as in Gaussian process regression and $V_{j}$ is the Voronoi cell of the *j*-th grid point $s^{\left[j\right]}$ given by $V_{j}:=\{s\in S\;|\;∥s-s^{\left[j\right]}∥\leq ∥s-s^{\left[i\right]}∥,\forall i\neq j\}.$ The predictive distribution of *z* given *y* and $\tilde{q}$ is thus given by

(8) $$\begin{array}{cc} \pi (z|\tilde{q},y) & =\sum_{q\in S}\pi \left(z|q,y\right)\pi \left(q|\tilde{q},y\right), \end{array}$$

where π(z|q,y) can be obtained by Equation (5) and the summation is over all possible locations in S.

**Figure 1:**
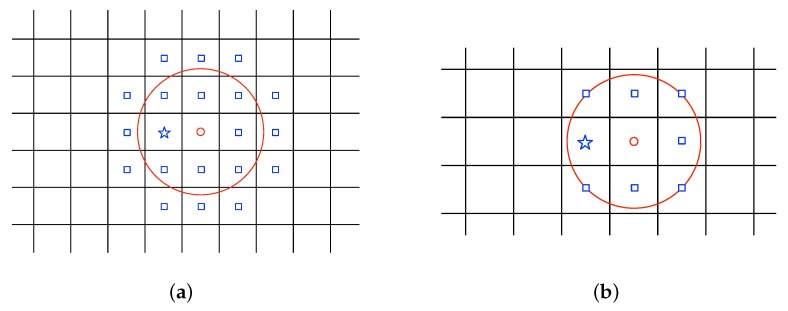
Example of localization uncertainty for $q^{\left[i\right]}$. The measured sampling location ${\tilde{q}}^{\left[i\right]}$ is indicated in a small red circle which is the closest point in the discrete support to the measured sampling position in the continuous space. The small red circle along with the blue squares and the blue star show the possible locations of the true sampling point $q^{\left[i\right]}$ according to the prior distribution $\pi (q^{\left[i\right]}|{\tilde{q}}^{\left[i\right]})$ with a compact support as shown in the big red circle. The blue star indicates $q^{\left[i\right]}$ which is the closest point in the discrete support to the true sampling position in the continuous space. (**a**) $h_{1}$; (**b**) $h_{2}$.

Figure 1 shows two examples of using this approximation approach with $h_{1}>h_{2}$ to convert a continuous space to a discrete one. When h→∞, $\tilde{q}\to {\tilde{q}}_{c}$ and the standard Gaussian process regression in a continuum space shall be recovered from the prediction using the GMRF in a discretized space.

## 3. Mobile Sensor Networks

Suppose that the sampling time $t\in Z_{>0}$ is discrete. Let $z_{t}:={\left({z_{t}}^{\left[1\right]},\cdots ,{z_{t}}^{\left[n\right]}\right)}^{T}\in R^{n}$ be the corresponding values of the scalar field at *n* special sites and time *t*.

Consider *N* spatially distributed mobile sensing agents indexed by j∈J:={1,⋯,N} sampling at time $t\in Z_{>0}$. At time *t*, agent *j* takes a noise corrupted measurement at its current location ${q_{t}}^{\left[j\right]}=s^{\left[i\right]}\in S$, i.e.,

(9) $$y_{t}^{\left[j\right]}=z_{t}^{\left[i\right]}+\epsilon_{t}^{\left[j\right]},\;\epsilon_{t}^{\left[j\right]}\overset{i.i.d.}{∼}N\left(0,\sigma_{\epsilon }^{2}\right),$$

where the measurement errors $\left\{\epsilon_{t}^{\left[j\right]}\right\}$ are assumed to be i.i.d. The measurement noise level $\sigma_{\epsilon }^{2}>0$ is assumed to be known. We denote all agents’ locations at time *t* by $q_{t}={\left({q_{t}}^{[1]T},\cdots ,{q_{t}}^{[N]T}\right)}^{T}\in S^{N}$ and the observations made by all agents at time *t* by $y_{t}={\left(y_{t}^{\left[1\right]},\cdots ,y_{t}^{\left[N\right]}\right)}^{T}\in R^{N}$. Furthermore, we denote the collection of agents’ locations and the collective observations from time 1 to *t* by $q_{1:t}={\left({q_{1}}^{T},\cdots ,{q_{t}}^{T}\right)}^{T}\in S^{Nt}$ and $y_{1:t}={\left(y_{1},\cdots ,y_{t}\right)}^{T}\in R^{Nt}$, respectively. In addition, let us define $z_{t}={\left({z_{t}}^{\left[1\right]},\cdots ,{z_{t}}^{\left[n\right]}\right)}^{T}\in R^{n}$ on S, and $\epsilon_{t}={\left(\epsilon_{t}^{\left[1\right]},\cdots ,\epsilon_{t}^{\left[N\right]}\right)}^{T}\in R^{N}$. We then have the following notation.

(10) $$y_{t}=H_{t}^{T}z_{t}+\epsilon_{t},$$

where $H_{\tau }\in R^{n\times N}$ is defined by

(11) $$H_{\tau }^{\left[ij\right]}=\left\{\begin{array}{cc} 1, & \mathrm{if}\;s^{\left[i\right]}=q_{\tau }^{\left[j\right]},\\ 0, & \mathrm{otherwise}. \end{array}\right.$$

## 4. Spatio-Temporal Field Model

The value of the scalar field at $s^{\left[i\right]}$, $z_{t}^{\left[i\right]}$ is modeled by a sum of a time-varying mean function and a GMRF

(12) $${z_{t}}^{\left[i\right]}=\lambda_{t}^{\left[i\right]}+\eta_{t}^{\left[i\right]},\;\forall i\in \left\{1,\cdots ,n\right\},\;t\in Z_{>0}.$$

Here the mean function $\lambda_{t}^{\left[i\right]}:S\times Z_{>0}\to R$ is defined as

(13) $$\lambda_{t}^{\left[i\right]}=f{\left(s^{\left[i\right]}\right)}^{T}\beta_{t},$$

where $f\left(s^{\left[i\right]}\right)={\left(f_{1}\left(s^{\left[i\right]}\right),\cdots ,f_{p}\left(s^{\left[i\right]}\right)\right)}^{T}\in R^{p}$ is a known regression function and $\beta_{t}={\left(\beta_{t}^{\left[1\right]},\cdots ,\beta_{t}^{\left[p\right]}\right)}^{T}\in R^{p}$ is an unknown vector of regression coefficients. The time evolution of $\beta_{t}\in R^{p}$ is modeled by a linear time-invariant system:

(14) $$\begin{array}{cc} \beta_{t+1} & =A_{t}\beta_{t}+B_{t}\omega_{t}, \end{array}$$

where $\omega_{t}∼N\left(0,W\right)$, $\beta_{0}∼N\left(\mu_{\beta_{0}},\Sigma_{\beta_{0}}\right)$, and $A_{t}$ and $B_{t}$ are known system parameters or can be found as discussed in [30].

In addition, we consider a zero-mean GMRF [31] $\eta_{t}={\left(\eta_{t}^{\left[1\right]},\cdots ,\eta_{t}^{\left[n\right]}\right)}^{T}\in R^{n}$ whose covariance matrix is given by

(15) $$E\left(\eta_{t}\eta_{k}^{T}|\theta \right)=Q_{\theta }^{-1}\delta \left(t-k\right),$$

where *t*, and *k* are time indices, and δ(·) is the Kronecker delta defined by

(16) $$\delta \left(k\right)=\left\{\begin{array}{cc} 1, & k=0,\\ 0, & \mathrm{otherwise}, \end{array}\right.$$

and the inverse covariance matrix (or precision matrix) $Q_{\theta }\in R^{n\times n}$ is a function of the hyperparameter vector θ.

There are different parameterizations of the GMRF (i.e., the precision matrix $Q_{\theta }$) [31]. Our Bayesian approach does not depend on the choice of the parameterization for the precision matrix. However, for a concrete and useful exposition, we describe a specific parameterization used in this paper. The precision matrix is parameterized with the full conditionals as follows. Let η be a GMRF on a regular two-dimensional lattice. The associated Gaussian full conditional mean is

(17) $$E\left(\eta_{t}^{\left[i\right]}|\eta_{t}^{\left[-i\right]},\theta \right)=-\frac{1}{Q_{\theta }^{\left[ii\right]}}\sum_{j=1}^{n}Q_{\theta }^{\left[ij\right]}\eta_{t}^{\left[j\right]},$$

where $Q_{\theta }^{\left[ij\right]}$ is the *i*-th row and *j*-th column element of $\kappa^{-1}Q_{\theta }$. Here, $\eta_{t}^{\left[-i\right]}$ is the collection of $\eta_{t}$ values everywhere except $s^{\left[i\right]}$. The hyperparameter vector is defined as $\theta ={\left(\kappa ,\alpha \right)}^{T}\in R_{>0}^{2}$, where α=a−4. The value of $Q_{\theta }^{\left[ii\right]}$ is $4+a^{2}$ as denoted at the center node of the graph. That of $Q_{\theta }^{\left[ij\right]}$ is −2a if *j* is one of the four closest neighbors of *i* in the vector 1-norm sense. Thus, the value of $Q_{\theta }^{\left[ij\right]}$ is zero if *j* is not one of the twelve closest neighbors of *i* (or twelve neighbors whose 1-norm distance to the *i*-th location is less than or equal to 2), as shown in [32]. The equation in Equation (17) states that the conditional expectation of $\eta_{t}^{\left[i\right]}$ given the value of $\eta_{t}$ everywhere else (i.e., $\eta_{t}^{\left[-i\right]}$) can be determined just by knowing the value of $\eta_{t}$ on the twelve closest neighbors (see more details in [32]). The resulting GMRF accurately represents a Gaussian random field with the Matérn covariance function as shown in [32]

(18) $$G\left(r\right)=\sigma_{f}^{2}\frac{2^{1-\rho }}{\Gamma (\rho )}{\left(\frac{\sqrt{2\rho }r}{\ell }\right)}^{\rho }K_{\rho }\left(\frac{\sqrt{2\rho }r}{\ell }\right),$$

where $K_{\rho }\left(\cdot \right)$ is a modified Bessel function [33], with order ρ=1, a bandwidth $\ell =1/h\sqrt{\frac{\alpha }{2}}$, and vertical scale $\sigma_{f}^{2}=1/4\pi \alpha \kappa$. The hyperparameter α>0 guarantees the positive definiteness of the precision matrix $Q_{\theta }$. In the case where α=0, the resulting GMRF is a second-order polynomial intrinsic GMRF [31,34].

From the presented model in Equations (12), (14), and (15), the distribution of $z_{t}$ given $\beta_{t}$ and θ is

(19) $$z_{t}|\beta_{t},\theta ∼N\left(F_{s}\beta_{t},Q_{\theta }^{-1}\right),$$

where $F_{s}:={\left(f\left(s^{\left[1\right]}\right),\cdots ,f\left(s^{\left[n\right]}\right)\right)}^{T}\in R^{n\times p}$.

In other words, $z_{t}|\beta_{t},\theta ∼\mathrm{GP}\left(F_{s}\beta_{t},\Sigma_{\theta }\right)\in R^{n}$ is a non-zero mean Gaussian process. Here, the covariance matrix $\Sigma_{\theta }$ is defined as inverse of the precision matrix (i.e., $\Sigma_{\theta }=Q_{\theta }^{-1}$). Note that the precision matrix is a positive definite matrix and invertible, and $\Sigma_{\theta }^{\left[ij\right]}=\mathrm{Cov}\left(z_{t}^{\left[i\right]},z_{t}^{\left[j\right]}\right)$, where $\Sigma_{\theta }^{\left[ij\right]}$ is the i,j-th element of the covariance matrix.

For simplicity, let us define $B_{t}=\left\{\beta_{t},q_{t},y_{t},\theta \right\}$. Using Gaussian process regression, the posterior distribution for $z_{t}|B_{t}\in R^{n}$ is given by

(20) $$\begin{array}{cc} \mu_{z_{t}|B_{t}}=\; & F_{s}\beta_{t}+\Sigma_{\theta }H_{t}{\left(H_{t}^{T}\Sigma_{\theta }H_{t}+\sigma_{\epsilon }^{2}I\right)}^{-1}\left(y_{t}-H_{t}^{T}F_{s}\beta_{t}\right),\\ \Sigma_{z_{t}|B_{t}}=\; & \Sigma_{\theta }-\Sigma_{\theta }H_{t}{\left(H_{t}^{T}\Sigma_{\theta }H_{t}+\sigma_{\epsilon }^{2}I\right)}^{-1}H_{t}^{T}\Sigma_{\theta }. \end{array}$$

The basic idea behind the model introduced in Equations (12), (14), and (15) stems from the space-time Kalman filter model proposed in [35]. The advantage of this spatio-temporal model with known hyperparameters is to make inferences in a recursive manner as the number of observations increases.

In this paper, however, uncertainties in the precision matrix and sampling positions are considered in a fully Bayesian manner. In contrast to [35,36], the GMRF with a sparse precision matrix is used to increase the computational efficiency.

## 5. Fully Bayesian Predictive Inference

### 5.1. Uncertain Hyperparameters and Exact Localization

In this section, we consider the problem of predicting a spatio-temporal random field, using successive noisy measurements sampled by a mobile sensor network. For a known covariance function, the prediction can be shown to be recursive [37] based on Gaussian process regression. The uncertainty in θ in a GMRF has been considered and its sequential prediction algorithms are derived in [12]. However, only the static field has been considered, i.e., $\mu_{t}=\mu_{0}$. In this section, we use a Bayesian approach to make predictive inferences of the spatio-temporal random field $z_{t}\in R^{n}$ for the case with uncertain hyperparameters and the exact localization. To this end, we use the following Assumptions 1–5.

**Assumption** **1.** *The spatio-temporal random field is generated by Equations* (12), (14), *and* (15)*;*

**Assumption** **2.** *The precision matrix $Q_{\theta }$ is a given function of an uncertain hyperparameter vector θ;*

**Assumption** **3.** *The noisy measurements $\left\{y_{t}\right\}$, as in Equation* (10)*, are continuously collected by mobile robotic sensors in time t;*

**Assumption** **4.** *The sample positions $\left\{q_{t}\right\}$ are measured precisely by mobile robotic sensors in time t;*

**Assumption** **5.** *The prior distribution of the hyperparameter vector θ is discrete with a support $\Theta =\{\theta^{\left(1\right)},\cdots ,\theta^{\left(L\right)}\}$.*

A.1 and A.2 stem from the discretization of a Gaussian process as we described in Section 2. From the model in A.1, the zero-mean GMRF represents a spatial structure by assuming that the difference between the parametric mean function and the dynamical environmental process is governed by a relatively large time scale. A.3 is a standard assumption over the measured observations [36]. A.4 will be relaxed to A.6 in Section 5.2 to deal with localization uncertainty. A.5 is from the discretization of the hyperparameter vector to replace an integration with a summation over possible hyperparameters.

We denote the full latent field of dimension n+p by $x_{t}={\left({z_{t}}^{T},\beta_{t}^{T}\right)}^{T}$. Let’s define $D_{k:r}:=\left\{P_{k-1},q_{k:r},y_{k:r}\right\}$, where $P_{k}=\left\{\mu_{x_{k}|D_{1:k}},\Sigma_{x_{k}|D_{1:k}}\right\}\cup \left\{\pi \left(\theta |D_{1:k}\right)|\theta \in \Theta \right\}$, and $D_{0}$ is assumed to be known.

We formulate the first problem as follows.

**Problem** **1.** *Consider the Assumptions 1–5. Our problem is to find the predictive distribution, mean, and variance of $x_{t}$ conditional on $D_{1:t}$.*

We then summarize the intermediate steps to obtain the solution to Problem 1. In what follows, our results are presented in terms of lemmas and theorems. We provide proofs when they are not straightforward.

**Lemma** **1.** *Under Assumptions 1 and 2, the predictive distribution of $x_{t}$ conditional on the hyperparameter vector θ and the measurements $D_{1:t-1}$ is Gaussian wit* *h the following mean and precision matrix:*

(21) $$\begin{array}{cc} \mu_{x_{t}|\theta ,D_{1:t-1}} & =\left(\begin{array}{c} F_{s}\mu_{\beta_{t}|\theta ,D_{1:t-1}}\\ \mu_{\beta_{t}|\theta ,D_{1:t-1}} \end{array}\right),\\ Q_{x_{t}|\theta ,D_{1:t-1}} & =\left(\begin{array}{cc} Q_{\theta } & -Q_{\theta }F_{s}\\ -F_{s}^{T}Q_{\theta } & F_{s}^{T}Q_{\theta }F_{s}+\Sigma_{\beta_{t}|\theta ,D_{1:t-1}}^{-1} \end{array}\right), \end{array}$$

*where $\mu_{\beta_{t}|\theta ,D_{1:t-1}}=A_{t}\mu_{\beta_{t-1}|\theta ,D_{1:t-1}}$ denotes the expectation of $\beta_{t}$ conditional on θ and $D_{1:t-1}$ and $\Sigma_{\beta_{t}|\theta ,D_{1:t-1}}=A_{t}\Sigma_{\beta_{t-1}|\theta ,D_{1:t-1}}A_{t}^{T}+B_{t}WB_{t}^{T}$ denotes the associated estimation error covariance matrix.*

**Proof** **of Lemma 1.** It can be shown by the update using the prior precision matrix and the previous iteration as in [12] (see more details in Section 3 of [12]). ☐

For a given hyperparameter vector θ, Equation (21) provides the optimal prediction of the spatio-temporal field in time *t* using data up to time t−1.

The following lemma is used to compute the posterior distribution of θ, recursively.

**Lemma** **2.** *Under Assumptions 3 and 4, the posterior distribution of the hyperparameter vector θ can be obtained recursively via*

(22) $$\pi \left(\theta |D_{1:t}\right)\propto \pi \left(y_{t}|\theta ,D_{1:t-1},q_{t}\right)\pi \left(\theta |D_{1:t-1}\right),$$

*where the distribution of $y_{t}$ given $\left\{\theta ,D_{1:t-1},q_{t}\right\}$ is Gaussian with the following mean and variance:*

(23) $$\begin{array}{cc} \mu_{y_{t}|\theta ,D_{1:t-1},q_{t}} & =\Gamma_{q_{t}}^{T}\mu_{x_{t}|\theta ,D_{1:t-1}},\\ \Sigma_{y_{t}|\theta ,D_{1:t-1},q_{t}} & =\Gamma_{q_{t}}^{T}\Sigma_{x_{t}|\theta ,D_{1:t-1}}\Gamma_{q_{t}}+\sigma_{\epsilon }^{2}I, \end{array}$$

*where $\Gamma_{q_{t}}^{T}=\left[H_{t}^{T}\;\;\;0\right]\in R^{N\times (n+p)}$.*

**Proof** **of** **Lemma** **2.** The posterior distribution of θ given in Equation (22) is computed by applying Bayes’ rule on $\pi (\theta |y_{t},D_{1:t-1})$. The predictive statistics of $y_{t}|\theta ,D_{1:t-1}$ are straightforward results of using Equation (10). Note that $\pi (\theta |D_{1:t-1})$ is equal to $\pi (\theta |D_{1:t-1},q_{t})$. ☐

**Lemma** **3.** *Under Assumptions 1–4, the full conditional distribution of $x_{t}$ for a given hyperparameter vector and data up to time t is*

$$x_{t}|\theta ,D_{1:t}∼N\left(\mu_{x_{t}|\theta ,D_{1:t}},Q_{x_{t}|\theta ,D_{1:t}}^{-1}\right),$$

*where*

(24) $$\begin{array}{cc} Q_{x_{t}|\theta ,D_{1:t}}=\; & Q_{x_{t}|\theta ,D_{1:t-1}}+\sigma_{\epsilon }^{-2}\Gamma_{q_{t}}\Gamma_{q_{t}}^{T},\\ \mu_{x_{t}|\theta ,D_{1:t}}=\; & \mu_{x_{t}|\theta ,D_{1:t-1}}+\sigma_{\epsilon }^{-2}Q_{x_{t}|\theta ,D_{1:t}}^{-1}\Gamma_{q_{t}}\left(y_{t}-\Gamma_{q_{t}}^{T}\mu_{x_{t}|\theta ,D_{1:t-1}}\right). \end{array}$$

In order to keep computing the prediction error covariance matrix $Q_{x_{t}|\theta ,D_{1:t}}^{-1}$ alone, the Woodbury lemma could be used to reduce the computational load as follows:

(25) $$\begin{array}{c} Q_{x_{t}|\theta ,D_{1:t}}^{-1}=Q_{x_{t}|\theta ,D_{1:t-1}}^{-1}-Q_{x_{t}|\theta ,D_{1:t-1}}^{-1}\Gamma_{q_{t}}{\left(\sigma_{\epsilon }^{2}I+\Gamma_{q_{t}}^{T}Q_{x_{t}|\theta ,D_{1:t-1}}^{-1}\Gamma_{q_{t}}\right)}^{-1}\Gamma_{q_{t}}^{T}Q_{x_{t}|\theta ,D_{1:t-1}}^{-1}, \end{array}$$

where $Q_{x_{t}|\theta ,D_{1:t-1}}^{-1}$ can be computed with blockwise inversion using Equation (21),

$$Q_{x_{t}|\theta ,D_{1:t-1}}^{-1}=\left(\begin{array}{cc} Q_{\theta }^{-1}+F_{s}\Sigma_{\beta_{t}|\theta ,D_{1:t-1}}F_{s}^{T} & F_{s}\Sigma_{\beta_{t}|\theta ,D_{1:t-1}}\\ \Sigma_{\beta_{t}|\theta ,D_{1:t-1}}^{T}F_{s}^{T} & \Sigma_{\beta_{t}|\theta ,D_{1:t-1}} \end{array}\right).$$

The blockwise inversion needs to be updated only with $\Sigma_{\beta_{t}|\theta ,D_{1:t-1}}$.

The following theorem explicitly illustrates how the results of Lemmas 2 and 3 lead to the predictive statistics of $x_{t}$ under Assumptions 1–5, which will be the solution to Problem 1.

**Theorem** **1.** *Under Assumption 5, the predictive distribution of $x_{t}|D_{1:t}$ is given by*

(26) $$\pi \left(x_{t}|D_{1:t}\right)=\sum_{\theta \in \Theta }\pi \left(x_{t}|\theta ,D_{1:t}\right)\pi \left(\theta |D_{1:t}\right),$$

*where $\pi (\theta |D_{1:t})$ and $\pi (x_{t}|\theta ,D_{1:t})$ are given by Lemmas 2 and 3, respectively. The predictive mean and variance follow as*

(27) $$\begin{array}{cc} \mu_{x_{t}|D_{1:t}}= & \sum_{\theta \in \Theta }\mu_{x_{t}|\theta ,D_{1:t}}\pi \left(\theta |D_{1:t}\right),\\ \Sigma_{x_{t}|D_{1:t}}= & \sum_{\theta \in \Theta }\left[\Sigma_{x_{t}|\theta ,D_{1:t}}+\left(\mu_{x_{t}|\theta ,D_{1:t}}-\mu_{x_{t}|D_{1:t}}\right)\right.\left.{\left(\mu_{x_{t}|\theta ,D_{1:t}}-\mu_{x_{t}|D_{1:t}}\right)}^{T}\right]\pi \left(\theta |D_{1:t}\right). \end{array}$$

**Proof** **of Theorem 1:** The predictive mean and variance is obtained by marginalizing over the conditional distribution of θ given $D_{1:t}$. The marginal mean and variance are $E_{Y}\left(Y\right)=E_{Y}\left(E_{X}\left(Y|X\right)\right)$ and ${\mathrm{Var}}_{Y}\left(Y\right)=E_{X}\left(\mathrm{Var}\left(Y|X\right)\right)+{\mathrm{Var}}_{X}\left(E\left(Y|X\right)\right)$, where $E_{X}$ and ${\mathrm{Var}}_{X}$ denote the expectation and the variance with respect to the random variable *X*. Having $Y:=x_{t}|D_{1:t}$ and $X:=\theta |D_{1:t}$ completes the proof of Theorem 1. ☐

The optimal prediction of the spatio-temporal field $x_{t}|\theta ,D_{1:t-1}$ using predictive statistics of $x_{t-1}|\theta ,D_{1:t-1}$ is provided by Lemma 1. Lemma 3 provides the optimal estimator of $x_{t}|\theta ,D_{1:t}$, using predictive statistics of $x_{t}|\theta ,D_{1:t-1}$ which is given by Lemma 1. Using Lemmas 1 and 3 sequentially, we can update predictive statistics of $x_{t}|\theta ,D_{1:t}$ for known hyperparameters. Lemma 2 gives us the posterior distribution of θ based on the measured data. Finally, Theorem 1 provides the optimal Bayesian prediction of the spatio-temporal random field with a time varying mean function and uncertain hyperparameters by marginalizing θ over the conditional distribution of $\theta |D_{1:t}$.

The proposed solution to the formulated problem is summarized by Algorithm 1.

**Algorithm 1** Sequential Bayesian Predictive InferenceInitialization:
1:initialize $F_{s}$  2:for θ∈Θ, initialize $Q_{\theta }$, and compute $Q_{\theta }^{-1}$
At time $t\in Z_{>0}$, do:
1:obtain new observations $y_{t}$ at current locations $q_{t}$  2:find the map $\Gamma_{q_{t}}$ from $q_{t}$ to spacial sites S, and compute radial basis values $F_{q_{t}}$ in $q_{t}$.3:**for**θ∈Θ**do**4: predict $\mu_{x_{t}|\theta ,D_{1:t-1}}$ and $Q_{x_{t}|\theta ,D_{1:t-1}}$ using measurements up to time t−1, given by Equation (21).5: compute $\mu_{x_{t}|\theta ,D_{1:t}}$ and $Q_{x_{t}|\theta ,D_{1:t}}$ given by Equation (24).6: compute $\mu_{y_{t}|\theta ,D_{1:t-1},q_{t}}$ and $\Sigma_{y_{t}|\theta ,D_{1:t-1},q_{t}}$ by Equation (23).7:calculate $\pi (\theta |D_{1:t})$ given by Equation (22).8:**end for**9:compute the predictive mean and variance using Equation (27).

### 5.2. Uncertain Hyperparameters and Localization

In the previous section, we assumed that the localization data $q_{1:t}$ is exactly known. However, in practice, positions of sensor networks cannot be measured without noise. Instead, for example, there could be several probable possibilities inferred from the measured position. In this section, the proposed method in the previous section will be extended for the uncertain localization data.

In order to take into account the uncertainty in the sampling positions, we replace Assumption 4 with the following Assumption 6.

**Assumption** **6.** *The prior distribution $\pi (q_{t})$ is discrete with a support $\Omega \left(t\right)=\{q_{t}^{\left(k\right)}|k\in I\left(t\right)\}$, which is given at time t along with the corresponding measurement $y_{t}$. Here, I(t)={1,⋯,γ(t)} denotes the index in the support and γ(t) is the number of the probable possibilities for $q_{t}$.*

A straightforward consequence of Assumption 6 is that the prior distribution $\pi (q_{k:r})$ is discrete with a support $\Omega \left(k:r\right):=\prod_{g=k}^{r}\Omega (g)$. In addition, $I\left(k:r\right):=\prod_{g=k}^{r}I(g)$ denotes the index in the support Ω(k:r), and $\gamma \left(k:r\right):=\prod_{g=k}^{r}\gamma (g)$ is the number of the probable possibilities for $q_{k:r}$. Now we state the problem as follows.

**Problem** **2.** *Consider Assumptions 1–3, 5, and 6. Our problem is to find the predictive distribution, mean and variance of $x_{t}$ conditional on the prior $P_{0}$ and the measurements $y_{1:t}$.*

For the sake of conciseness, let us define $R_{r:k}:=\left\{P_{r-1},y_{r:k}\right\}$. We then have $R_{r:k}\subset D_{r:k}$, where we recall that $D_{r:k}:=\left\{P_{r-1},q_{r:k},y_{r:k}\right\}$.

To solve Problem 2, we first look for a way to compute the posterior distribution of $q_{t}$ as summarized in the following lemma.

**Lemma** **4.** *Consider $\pi (y_{t}|\theta ,D_{1:t-1}^{\left(n\right)},q_{t}^{\left(k\right)})$ given by Equation* (23) *and $\pi (\theta |D_{1:t-1}^{\left(n\right)})$ given by Equation* (22), *where n∈I(1:t−1), k∈I(t), and $D_{1:t-1}^{\left(n\right)}:=\left\{P_{0},q_{1:t-1}^{\left(n\right)},y_{1:t-1}\right\}$. Under Assumption 5, we have*

$$\pi \left(y_{t}|D_{1:t-1}^{\left(n\right)},q_{t}^{\left(k\right)}\right)=\sum_{\theta \in \Theta }\pi \left(y_{t}|\theta ,D_{1:t-1}^{\left(n\right)},q_{t}^{\left(k\right)}\right)\pi \left(\theta |D_{1:t-1}^{\left(n\right)}\right).$$

*Under Assumption 6, the posterior distribution of $q_{t}$ can be obtained, recursively, via*

(28) $$\begin{array}{cc} \pi \left(q_{1:t-1}^{\left(n\right)},q_{t}^{\left(k\right)}|R_{1:t}\right)\propto & \pi \left(q_{1:t-1}^{\left(n\right)}|R_{1:t-1}\right)\pi \left(y_{t}|D_{1:t-1}^{\left(n\right)},q_{t}^{\left(k\right)}\right)\pi \left(q_{t}^{\left(k\right)}\right). \end{array}$$

We now give the exact solution to Problem 2 as follows.

**Theorem** **2.** *Consider the predictive distribution $\pi (x_{t}|D_{1:t}^{\left(i\right)})$ given by Theorem 1 and the posterior $\pi \left(q_{1:t}^{\left(i\right)}|R_{1:t}\right)$ given by Lemma 4, where $q_{1:t}^{\left(i\right)}\in \Omega \left(1:t\right)$ and $D_{1:t}^{\left(i\right)}=\left\{P_{0},q_{1:t}^{\left(i\right)},y_{1:t}\right\}$. Under Assumption 6, the predictive distribution of $x_{t}|R_{1:t}$ can be obtained as follows:*

(29) $$\pi \left(x_{t}|R_{1:t}\right)=\sum_{i\in I(1:t)}\pi \left(x_{t}|D_{1:t}^{\left(i\right)}\right)\pi \left(q_{1:t}^{\left(i\right)}|R_{1:t}\right).$$

*Consequently, the predictive mean and variance are given by the formulas:*

(30) $$\begin{array}{cc} \mu_{x_{t}|R_{1:t}}= & \sum_{i\in I(1:t)}\mu_{x_{t}|D_{1:t}^{\left(i\right)}}\pi \left(q_{1:t}^{\left(i\right)}|R_{1:t}\right),\\ \Sigma_{x_{t}|R_{1:t}}= & \sum_{i\in I(1:t)}\left[\Sigma_{x_{t}|D_{1:t}^{\left(i\right)}}+\left(\mu_{x_{t}|D_{1:t}^{\left(i\right)}}-\mu_{x_{t}|R_{1:t}}\right)\right.\left.{\left(\mu_{x_{t}|D_{1:t}^{\left(i\right)}}-\mu_{x_{t}|R_{1:t}}\right)}^{T}\right]\pi \left(q_{1:t}^{\left(i\right)}|R_{1:t}\right). \end{array}$$

**Proof** **of Theorem 2:** The proof is similar to that of Theorem 1. Hence, the predictive mean and variance are obtained by marginalizing over the conditional distribution of $q_{1:t}$. The marginal mean and variance are $E_{Y}\left(Y\right)=E_{Y}\left(E_{X}\left(Y|X\right)\right)$ and ${\mathrm{Var}}_{Y}\left(Y\right)=E_{X}\left(\mathrm{Var}\left(Y|X\right)\right)+{\mathrm{Var}}_{X}\left(E\left(Y|X\right)\right)$, where $E_{X}$ and ${\mathrm{Var}}_{X}$ denote the expectation and the variance with respect to the random variable *X*. Having $Y:=x_{t}|R_{1:t}$ and $X:=q_{1:t}|R_{1:t}$ proves Theorem 2. ☐

The complexity of the proposed algorithm in Theorem 2 is proportional to the number of possibilities for $q_{1:t}$. The result of Lemma 4 enables us to compute these probable possibilities recursively. However, the number of the non-zero probable combinations grows exponentially by a power of time index *t*.

In what follows, we propose an approximation, with a controllable trade-off between approximation error and complexity, to the exact solution given in Theorem 2 by including an option of ignoring uncertainties on past position data. This approximation will include a case of the exact solution with the maximal and original complexity. The idea is based on the fact that the estimation of $x_{t}$ is more susceptible to the uncertainties in recently sampled positions as compared to old ones. To formulate our idea clearly, we present first the following results.

**Lemma** **5.** *Using prior distribution of $x_{t-m}$ and measured data $y_{t-m+1:t}$, where $m\in Z_{>0}$, the posterior distribution of $q_{t-m+1:t}$ can be obtained recursively via*

(31) $$\begin{array}{cc} \pi & \left(q_{t-m+1:t-1}^{\left(j\right)},q_{t}^{\left(k\right)}|R_{t-m+1:t}\right)\propto \pi \left(q_{t-m+1:t-1}^{\left(j\right)}|R_{t-m+1:t-1}\right)\pi \left(y_{t}|D_{t-m+1:t-1}^{\left(j\right)},q_{t}^{\left(k\right)}\right)\pi \left(q_{t}^{\left(k\right)}\right), \end{array}$$

*where j∈I(t−m+1:t−1), and k∈I(t).*

**Theorem** **3.** *Consider $\mu_{x_{t}|D_{t-m+1:t}^{\left(h\right)}}$ and $\Sigma_{x_{t}|D_{t-m+1:t}^{\left(h\right)}}$ computed by Theorem 1. Under Assumption 6, the predictive statistics of $x_{t}|R_{t-m+1:t}$ are as follows:*

(32) $$\begin{array}{cc} \mu_{x_{t}|R_{t-m+1:t}}= & \sum_{h\in I(t-m+1,t)}\mu_{x_{t}|D_{t-m+1:t}^{\left(h\right)}}\pi \left(q_{t-m+1:t}^{\left(h\right)}|R_{t-m+1:t}\right),\\ \Sigma_{x_{t}|R_{t-m+1:t}}= & \sum_{h\in I(t-m+1,t)}\left[\Sigma_{x_{t}|D_{t-m+1:t}^{\left(h\right)}}+\left(\mu_{x_{t}|D_{t-m+1:t}^{\left(h\right)}}-\mu_{x_{t}|R_{t-m+1:t}}\right)\right.\\ & \left.{\left(\mu_{x_{t}|D_{t-m+1:t}^{\left(h\right)}}-\mu_{x_{t}|R_{t-m+1:t}}\right)}^{T}\right]\pi \left(q_{t-m+1:t}^{\left(h\right)}|R_{t-m+1:t}\right). \end{array}$$

To implement approximations to the predictive statistics of $x_{t}|R_{1:t}$ which are given by Theorem 2, we consider the following conditions.

**C.1**  For 1≪m≤t, we have that
  (33) $$\pi \left(x_{t}|R_{1:t}\right)\approx \pi \left(x_{t}|R_{t-m+1:t}\right).$$
**C.2**  For 1≪m≤t, $P_{t}$ can be approximated by
  (34) $$\begin{array}{cc} P_{t}\approx & \left\{\mu_{x_{t}|R_{t-m+1:t}},\Sigma_{x_{t}|R_{t-m+1:t}}\right\}\cup \left\{\pi \left(\theta |R_{t-m+1:t}\right)|\theta \in \Theta \right\}. \end{array}$$

Under conditions C.1 and C.2, it is natural for us to propose the following approximations:

(35) $$\begin{array}{c} \mu_{x_{t}|R_{1:t}}\approx \mu_{x_{t}|R_{t-m+1:t}},\;\Sigma_{x_{t}|R_{1:t}}\approx \Sigma_{x_{t}|R_{t-m+1:t}}. \end{array}$$

In Theorem 3, the predictive statistics of $x_{t}|D_{t-m+1:t}^{\left(h\right)}$ are obtained from Algorithm 1, which is given in Section 5.1. The only difference is that we start from time t−m+1 instead of time 1 with $P_{t-m}$ instead of $P_{0}$. Note that, without condition C.2, we cannot use Algorithm 1 to calculate the statistics of $x_{t}|D_{t-m+1:t}^{\left(h\right)}$. The proposed approximation for the case of uncertain localization in Equation (35) is quite different from a mere truncation of old data in the sense that past measurements still affect the current estimation through the approximately updated prior information using Equation (34). Note that we update the prior information from $P_{t-m}$ to $P_{t}$ with the cumulative data collected from time t−m+1 up to time *t*, which is different from only using truncated observations. The proposed approximation for the formulated problem is summarized by Algorithm 2.

To further understand the nature of the proposed approximation, consider the following two extreme special cases.

**Corollary** **1.** *As a special case of Theorem 3 for m=1, the posterior distribution of $q_{t}$ can be obtained via*

(36) $$\pi \left(q_{t}^{\left(k\right)}|P_{t-1},y_{t}\right)\propto \pi \left(y_{t}|P_{t-1},q_{t}^{\left(k\right)}\right)\pi \left(q_{t}^{\left(k\right)}\right),$$

*where k∈I(t). The predictive distribution of $x_{t}|R_{1:t}$ can be approximated in a constant time as time t increases in a sequential way.*

$$\pi \left(x_{t}|R_{1:t}\right)\approx \pi \left(x_{t}|P_{t-1},y_{t}\right),$$

*where*

(37) $$\begin{array}{cc} \pi \left(x_{t}|P_{t-1},y_{t}\right)= & \sum_{k\in I(t)}\pi \left(x_{t}|P_{t-1},q_{t}^{\left(k\right)},y_{t}\right)\pi \left(q_{t}^{\left(k\right)}|P_{t-1},y_{t}\right) \end{array}$$

*and the posterior $\pi \left(q_{t}^{\left(k\right)}|P_{t-1},y_{t}\right)$ is given by Equation* (36). *Consequently, the predictive mean $\mu_{x_{t}|R_{1:t}}$ and variance $\Sigma_{x_{t}|R_{1:t}}$ can be approximated by $\mu_{x_{t}|P_{t-1},y_{t}}$ and $\Sigma_{x_{t}|P_{t-1},y_{t}}$, respectively, i.e.,*

(38) $$\begin{array}{cc} \mu_{x_{t}|P_{t-1},y_{t}}\;= & \sum_{k\in I(t)}\mu_{x_{t}|t-1,q_{t}^{\left(k\right)},y_{t}}\pi \left(q_{t}^{\left(k\right)}|P_{t-1},y_{t}\right),\\ \Sigma_{x_{t}|P_{t-1},y_{t}}= & \sum_{k\in I(t)}\left[\Sigma_{x_{t}|P_{t-1},q_{t}^{\left(k\right)},y_{t}}+\left(\mu_{x_{t}|P_{t-1},q_{t}^{\left(k\right)},y_{t}}-\mu_{x_{t}|P_{t-1},y_{t}}\right)\right.\\ & \left.{\left(\mu_{x_{t}|P_{t-1},q_{t}^{\left(k\right)},y_{t}}-\mu_{x_{t}|P_{t-1},y_{t}}\right)}^{T}\right]\pi \left(q_{t}^{\left(k\right)}|P_{t-1},y_{t}\right). \end{array}$$

**Corollary** **2.** *For another special case with m=t, Theorem 3 becomes Theorem 2.*

For a fixed $m\in Z_{>0}$, Algorithm 2 is scalable as time *t* increases. In our approach, the level of the approximation can be controlled by users by selecting a trade-off (or by choosing *m*) between the approximation error and complexity. Most simplistic and practical approximation can be obtained by choosing m=1 as in Corollary 1. The original exact solution with maximal complexity is recovered by selecting m=t as shown in Corollary 2.

**Algorithm 2** Sequential Bayesian Predictive Inference Approximation with Uncertain Localization
At time $t\in Z_{>0}$, do:
1:obtain new observations $y_{t}$ along with the probabilities for locations $\pi (q_{t})$2:**for**$q_{t}^{\left(h\right)}\in \Omega \left(t-m+1:t\right)$**do**3: predict $\mu_{x_{t}|D_{t-m+1:t}^{\left(h\right)}}$, $\Sigma_{x_{t}|D_{t-m+1:t}^{\left(h\right)}}$ and $\pi (y_{t}|D_{t-m+1:t-1}^{\left(j\right)},q_{t}^{\left(k\right)})$ using Algorithm 14: compute $\pi \left(q_{t-m+1:t}^{\left(h\right)}|R_{t-m+1:t}\right)$ by Lemma 5.5:**end for** 6:compute $\mu_{x_{t}|R_{t-m+1:t}}$ and $\Sigma_{x_{t}|R_{t-m+1:t}}$, using Theorem 3.  7:use following approximation to update estimations
$$\mu_{x_{t}|R_{1:t}}\approx \mu_{x_{t}|R_{t-m+1:t}},\;\Sigma_{x_{t}|R_{1:t}}\approx \Sigma_{x_{t}|R_{t-m+1:t}},$$
$$P_{t}\approx \{\mu_{x_{t}|R_{t-m+1:t}},\Sigma_{x_{t}|R_{t-m+1:t}},\pi \left(\Theta |R_{t-m+1:t}\right)\}.$$

### 5.3. Complexity of Algorithms

In this section, we discuss complexity aspects of the proposed algorithms. For a fixed number of the radial basis functions (i.e., *p*) and a fixed number of the special sites (i.e., *n*), the computational complexity of Algorithm 1 is dominated by Equation (24). The complexity of Algorithm 1 in each time step is $O(LN^{2})$, where *L* is the number of possible hyperparameter vectors and *N* is the number of agents. The complexity of Algorithm 2 in time *t* is $O\left(\gamma (t-m+1:t)\right)$ times the complexity of Algorithm 1 for *m* time steps. Hence, the complexity of Algorithm 2 in time *t* is $O\left(\gamma \left(t-m+1:t\right)LN^{2}M\right)$. The numbers of special sites and radial basis functions affect the complexity of Algorithm 1 as well. The complexity of the three cases is $O(n^{3})$ with respect to the number of special sites due to the matrix inversion. Thus, if we use finer grids, we increase the number of special sites, i.e., *n*, and the complexity increase cubical. For a fixed set of *L*, *n* and *N*, the complexity of Algorithm 1 with respect to *p* is $O(p^{3})$.

## 6. Experimental Results

**Figure 2:**
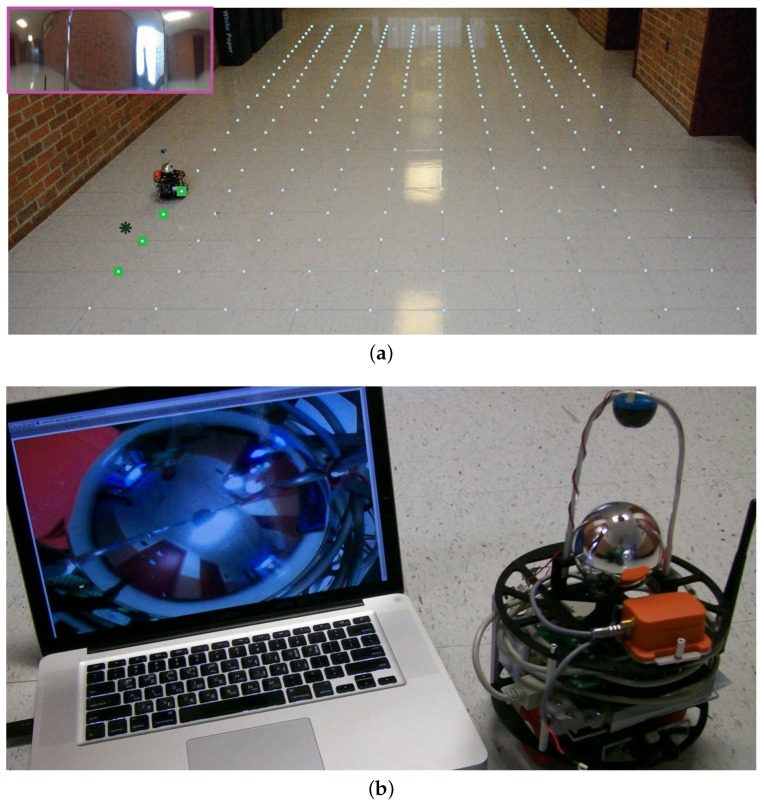
The experimental setup. (**a**) The measured position of the robot and four possible sampling positions are shown in dark green star (*) and light green squares (□), respectively. The spatial sites are marked with aqua blue dots on the ground. The panoramic view of the robot is pictured on the upper left-hand side of the figure; (**b**) a vision-based robot is built with a 360-degree omnidirectional camera.

Similarly to [38], a vision-based robotic sensor was built to validate the proof of concept. The wireless mobile robot is equipped with two motorized wheels, a micro-controller, and a 360-degree omnidirectional camera, a motion sensor, a wireless receiver, and a transmitter. The omnidirectional camera is homemade from a cheap Wi-Fi remote CCD camera (Ai-Ball^®^, Trek 2000 International, Singapore) and a globe mirror. The vision images of the 360-degree environment around the robot produced by the omnidirectional camera are streamed via 802.11 b/g Wi-Fi interface to a laptop for image processing (see Figure 2).

In this section, we apply the proposed prediction algorithms to real experimental data. Figure 2 shows our experimental setup in which a redness intensity field is sampled by the captured images from the CCD camera on top of the mobile robot. The CCD camera captures 360-degree images. The redness intensity is computed by simply averaging the red component of the RGB picture. Noisy visual measurements are sampled at random sampling positions by our robot. The position of the robot has been measured by an image processing software which is built by the authors and the true sampling positions are obtained manually by inspection.

The experimental objective is to predict the redness intensity field over a spatial space or a spatio-temporal space using the scalar field model proposed in Section 4. Note that each image has a lot of information. However, we only used the redness out of each image as a scalar value of interest. In the future, we plan to extend this experiment for multivariate random fields for multiple features from each image. Here we are considering two different scenarios. First, we consider a static field and then a time-varying field with a moving person in the surveillance region.

### 6.1. Spatial Field in the Static Experiment

In this study, the spatial sites in S are considered to be 10×26 grid points, i.e., n=260. The grid points are shown in Figure 2 with aqua-blue dots. The mean function $\mu_{t}$ consists of only one radial basis function that keeps moving average of the field. Note that this basis function can model the changes in the brightness of the images caused by the slow changes of the environment lights. The center of the radial basis function is (5,13), and its bandwidth $\sigma_{1}$ is ∞. The prior distribution of the hyperparameter vector θ is chosen to be discrete with a support Θ={25,50,100,200,400}×{0.1,0.2,0.4,0.8,1.6} and the associated uniform probabilities. The measurement noise variance $\sigma_{\epsilon }=0.01$ is estimated.

**Figure 3:**
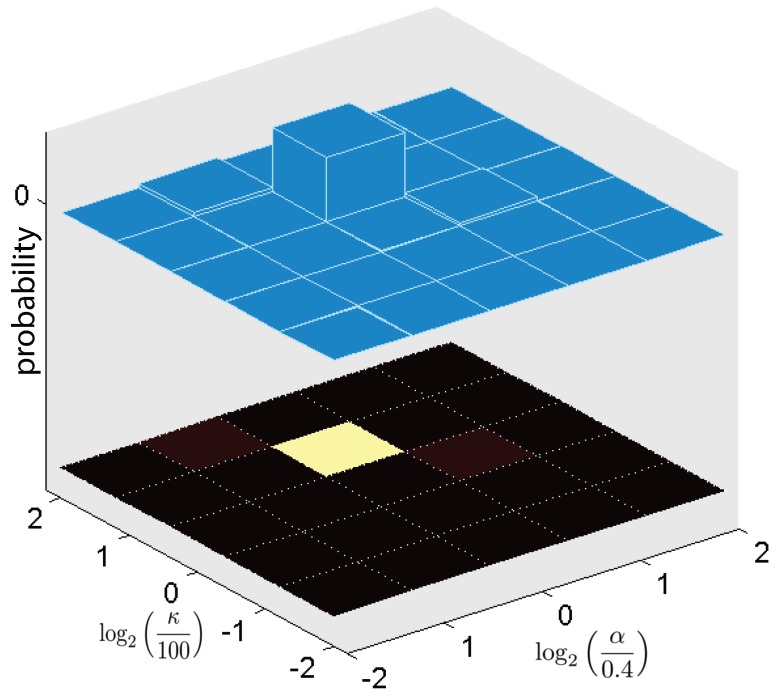
The posterior probability of the hyperparameter vector at t=2.

To demonstrate the usefulness of our model in Equations (12), (14), and (15), and our prediction algorithm, we simulate eighty mobile robots with a single mobile robot that measures spatially distributed eighty samples, i.e., n=80, where each of nine sampling positions is uncertain with four possibilities. Thus, there is $4^{9}$ possible combinations for this set of sampling positions. After two sets of observations, the resulting posterior probabilities of the hyperparameters for Case 1 are shown in Figure 3. Figure 3 shows that the estimated hyperparameters converge to κ=100 and α=0.8, which is equivalent to ℓ=1.58 and $\sigma_{f}=0.0315$. 

**Figure 4:**
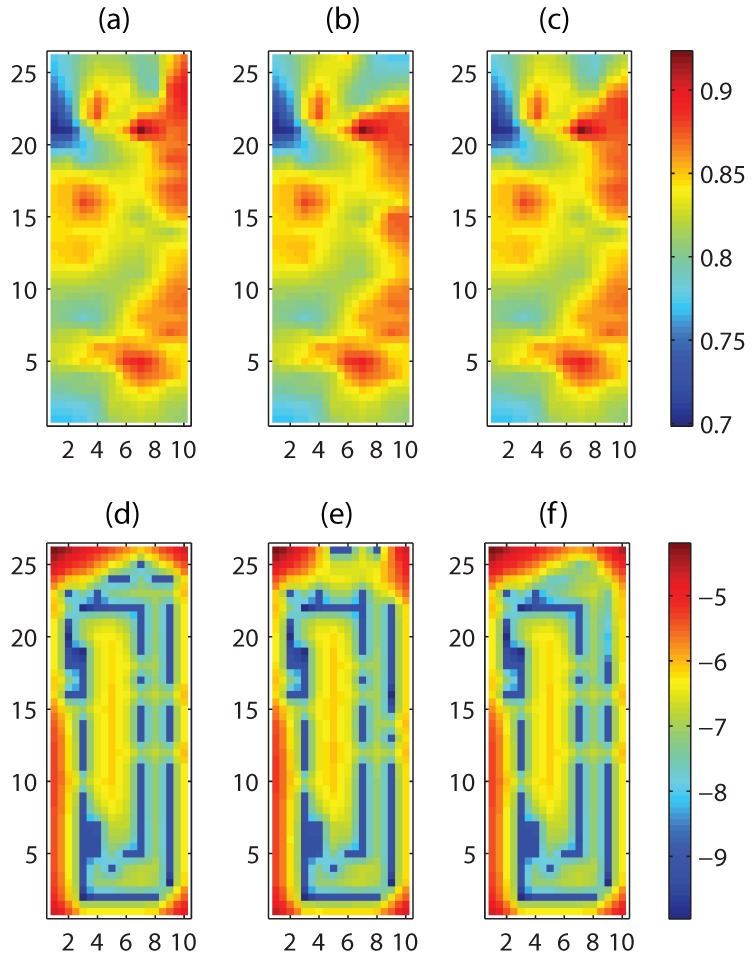
The prediction results of Cases 1, 2, and 3 at time t=2 are shown in the first, second, and third columns, respectively. The first and second rows correspond to the predictions and the natural logarithm of the prediction error variance.

We consider three cases: Case 1 using Algorithm 1 with exact sampling positions, Case 2 using Algorithm 1 naively to measured sample positions including noisy locations, and Case 3 using by applying Algorithm 2 with m=1. The prediction and prediction error variance are computed for Cases 1, 2, and 3. The prediction and the prediction error variance using true sampling positions (Case 1), are shown in Figure 4a,d, respectively. Red and blue colors represent the highest and the lowest values, respectively. Figure 4b,e show the resulting fields, by applying Algorithm 1 naively to measured sample positions including noisy locations (Case 2).

Figure 4c,f show the results by applying Algorithm 2 with m=1 (Case 3). Blue trails shown in Figure 4d–f represent the low predicted error variances due to sampling. The results confirm that the quality of the prediction in Case 3 are not very compromised as compared to Case 1 and demonstrate the capability of our proposed algorithm to deal with uncertain sampling positions.

**Figure 5:**
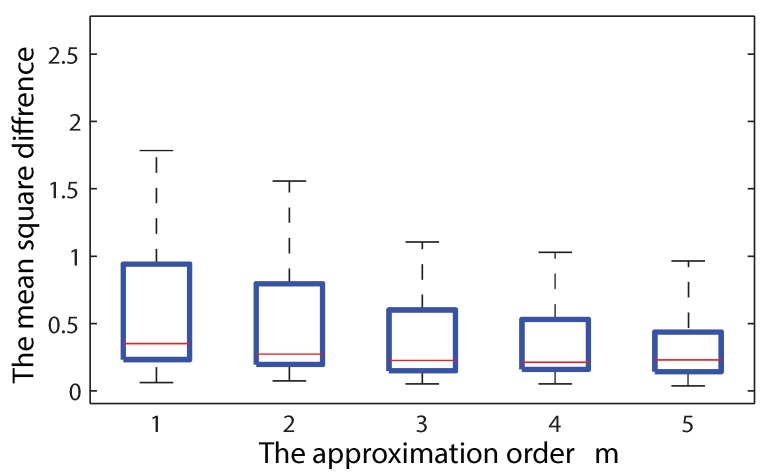
The mean square difference between Case 1 and Case 3 is shown for the different approximation orders m=1,⋯,5. On each box, the central mark is the median, the upper and lower edges of the box are the 25th and 75th percentiles, respectively. The whiskers extend to the most extreme data points.

Figure 5 shows the controllable trade-off between approximation error and complexity for another simulated example. The complexity of Algorithm 2 increases exponentially with respect to *m*. The predicted field is compared between Case 1, which is the best prediction quality expected, and Case 3. Clearly, by increasing *m*, the mean square difference between Case 1 and Case 3 decreases. However, this mild improvement results in the exponentially increasing computational load, which guides us to use m=1 for practical cases.

**Figure 6:**
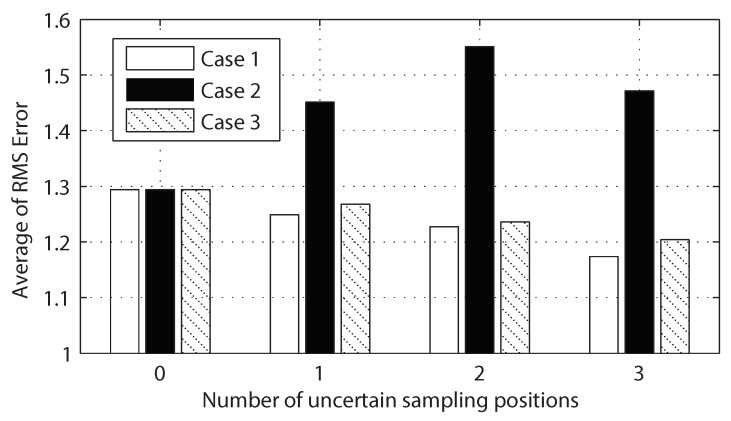
The number of observations with uncertain sampling position on the three different cases is shown on the horizontal axis. The field prediction root mean square (RMS) error for Case 1, Case 2 and Case 3 are shown with white, black, and hatched bars, respectively.

Figure 6 shows the effect of an increasing number of observations with uncertain sampling positions on the three cases from the same simulated example. Here we assume that we have seven observations with known true sampling positions plus a few observations with uncertain sampling positions. Clearly, the root mean square (RMS) error decreases in Case 1 and Case 3 by adding new observations with true and uncertain sampling positions, respectively. On the other hand, as shown in Figure 6, adding new observations with noisy sampling positions could increase RMS error for Case 2. Figure 6 also shows the efficacy of our proposed algorithm, which can be compared to the previous work [23]. The previous work in [23] was limited to a static random field and achieved 3.63 RMS error with a bandwidth of 4.47. Note that our proposed method (Case 3) considers the temporal random field and achieved averaged RMS error less than 1.2 with a bandwidth of 1.58. For a fair comparison against [23], we normalize the input space of the GMRF by the bandwidth and compare resulting values in terms of RMS over bandwidth. Our current work achieved 0.759 RMS/bandwidth outperforming the previous work in [23] with 0.812 RMS/bandwidth.

### 6.2. Spatio-Temporal Field in the Dynamic Experiment

**Figure 7:**
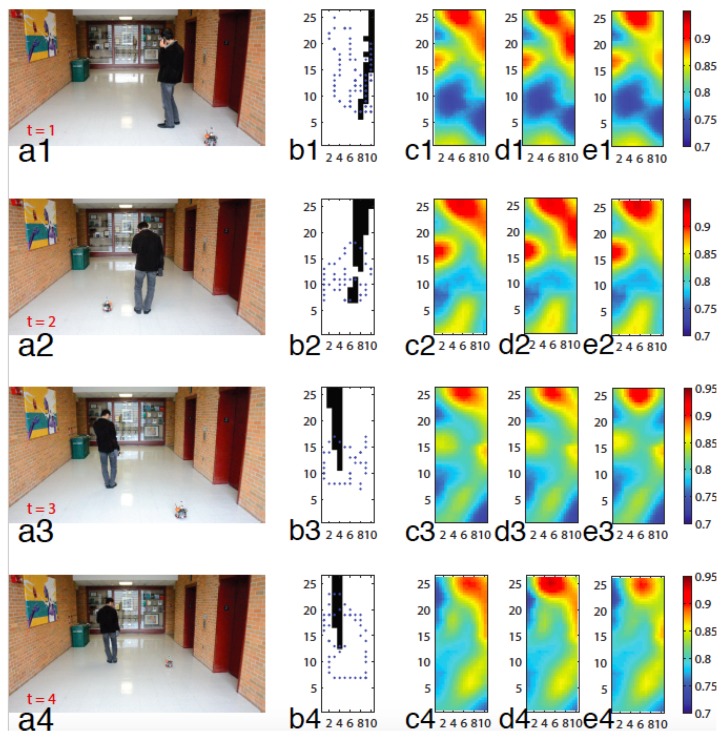
The time varying field is shown for four time iterations. The first column (a1–4) shows the moving object in the field. The second column (b1–4) shows the sampling positions with blue dots and the positioning blind spot with black areas. The third (c1–4), fourth (d1–4) and fifth (e1–4) columns show predicted field for Case 1, Case 3 and Case 4, respectively. The rows are correspond to the time intervals t=1,2,3 and 4.

In this scenario, the spatial sites are the same as in the previous experiment. The mean function $\mu_{t}$ consists of twenty nine radial basis functions. The centers of radial basis functions are {(5,13)}∪{1,4,7,10}×{1,5,9,13,17,21,25}. The time evolution of $\beta_{t}$ is modeled by Equation (14), where the state matrix $A_{t}$ and the input matrix $B_{t}$ are given by *I* and 0.05I, respectively. The first radial basis function has an infinity bandwidth (i.e., $\sigma_{1}=\infty$) to represent the average of the field, and the others have a bandwidth that is equal to $\sigma_{j}=4$. A person moves to a spot and stays still while a robot is collecting observations, the process of which is then repeated in order to efficiently simulate multiple robots. The robot collects 57, 40, 31, and 45 observations corresponding to times t=1,2,3, and 4, respectively. The first column (a1–4) in Figure 7 shows the positions of moving robot and person in the domain. As mentioned, the position of the robot has been measured with a fixed camera and an image processing software. Sometimes, the robot is not visible by the positioning system since the moving person blocks the robot. The black areas in the second column (b1–4) of Figure 7 show the blind spots of the positioning system. Note that other positioning systems like GPS also have blind spots such as GPS denied areas. Therefore, when the robot moves to a blind spot the position cannot be determined precisely. In this case, we assign different probabilities on multiple sampling positions.

True sampling positions in each time step are shown with blue dots in the second column (b1–4) of Figure 7 while just blue dots in the white area are measured through the positioning system and the positions in the blind spots are recorded manually for a comparison purpose.

The prediction results of Case 1 and Case 3 are compared with a trivial method of prediction defined as follows.

Case 4: The fifth column (e1–4) of Figure 7 shows the resulting fields, by applying Algorithm 1 on just observed sampling positions. Here, all the observations whose sampling positions are uncertain are discarded. In particular, 12,3,2, and 4 observations have been discarded for time t=1,2,3 and 4, respectively.

The predicted field using true sampling positions (Case 1) and uncertain sampling positions (Case 3) are shown in the third (c1–4) and forth (d1–4) columns of Figure 7, respectively. Since in the fully automated experiment true sampling positions in the blind spot are not available, we used the proposed algorithm in this paper to deal with uncertain sampling positions in the blind spot areas. The predicted field simply by discarding uncertain sampling positions is shown in the fifth column (e1–4) of Figure 7. The results obtained for Case 3 with m=1, is not compromised considering the result for Case 1. Thus, the experimental result demonstrates the effectiveness of the proposed algorithm.

## 7. Conclusions

We have tackled a problem of predicting a spatio-temporal field using successive noisy scalar measurements obtained by mobile robotic sensors, some of which have uncertain localization. We developed the spatio-temporal field of interest using a GMRF and designed sequential prediction algorithms for computing the exact and approximated predictive inference from a Bayesian point of view. The most important contribution is that the computation times for Algorithms 1 and 2 with a finite *m* at each time step do not grow as the number of measurements increases. Two different static and time-varying experimental results along with a comparison study using simulation results provide a solid proof of concept on the proposed scheme. The proposed algorithm will be useful for robotics applications such as environmental monitoring by autonomous aquatic robots and drones. Future work is to apply our algorithms to spatio-temporal sensory information fusion for autonomous driving. In particular, we plan to predict a spatio-temporal scalar field of a risk measure. The self-driving vehicle will be designed to perform path-planning taking into account such predicted risk measures over space and time for better safety.
